# Assessment of attachment disorder symptoms in foster children: comparing diagnostic assessment tools

**DOI:** 10.1186/s13034-018-0250-3

**Published:** 2018-08-17

**Authors:** Josephine D. Kliewer-Neumann, Janin Zimmermann, Ina Bovenschen, Sandra Gabler, Katrin Lang, Gottfried Spangler, Katja Nowacki

**Affiliations:** 1 University of Applied Sciences and Arts, Dortmund, Germany; 2 University of Erlangen-Nuremberg, Erlangen, Germany

**Keywords:** Reactive attachment disorder (RAD), Disinhibited social engagement disorder (DSED), Diagnosis, Foster care, Assessment

## Abstract

**Background:**

Standardized methods for assessing attachment disorders are scarce but needed for research and practice.

**Methods:**

In the current study, several assessments for attachment disorder symptoms are used within a German sample of foster children after being exposed to neglect and maltreatment in their biological families. The symptoms were assessed with four established assessment methods based on both parents’ report and behavioral observation: The Rating for Infant Stranger Engagement, the Stranger at the Door, the Disturbances of Attachment Interview and the Reactive Attachment Disorder Questionnaire.

**Results:**

The foster care sample showed symptoms of both the inhibited and the disinhibited attachment disorder. The degree of symptoms is comparable to previous findings. The results of the different tools investigating the disinhibited type of attachment disorder are correlated to each other, but do not overlap.

**Conclusions:**

Although all approaches are based on the clinical criteria of the DSM-IV, the assessments do not coincide. Each tool provides a different point of view on the symptoms, so a multi methodical approach for assessing attachment disorder symptoms should be implemented. Furthermore, the inhibited and the disinhibited symptoms represent separate categories, as reflected in the DSM-5, requiring separate assessment.

## Background

Inadequate care like maltreatment, neglect or severe deprivation in terms of no consistent caregiver, is known leading to behavior that can be diagnosed as attachment disorders. Studies focusing on children raised under extreme conditions of caregiving, like the first major longitudinal study by Tizard and Rees [1] found deviant social behavior within a group of children raised in institutions. Most of these children showed emotional withdrawal, and unresponsiveness or indiscriminate behavior, friendliness and, overly familiar behavior. These two behavioral patterns provided the foundation for picturing reactive attachment disorders in the DSM-III for the first time. Later studies of children who experienced institutional care in Romanian orphanages identified similar disorder symptoms [2–4]. The criteria for attachment disorders have been revised several times and the recent DSM-5 divides the reactive attachment disorder (RAD), referring to the inhibited symptoms, and the disinhibited social engagement disorder (DSED), referring to the disinhibited symptom pattern.

### Etholgy and risk factors

Recently considerable research on disturbances of attachment has been done, but there is little empirical data regarding the prevalence of the disorders [5]. Both RAD and DSED are exceedingly rare in low-risk samples and occur in a minority of children, raised under extreme conditions [6]. In a sample of 300 preschoolers aged between 2 and 5 years, no child met the diagnostic criteria for an attachment disorder [7]. Minnis et al. [8] found a prevalence of 1.4% in a deprived population of school-aged children and Gleason et al. [9] reported 4.11% of RAD and 20% of DSED in a population of previously institutionalized children. Both DSM and ICD describe poor caregiving as the core factor for the development of attachment disorder symptoms [10]. Hall and Geher [11] describe positive caregiver child interaction leading to bonding and attachment, whereas the absence may lead to attachment disorder symptoms. Several studies showed significant correlations between institutional care and attachment disorder symptoms [2–4]. It has been shown that attachment disorder behaviors are linked to the duration of deprivation [3], sensitivity of the environment and quantity of caregivers [4]. Thus, the less sensitive an environment is the more common attachment disorder symptoms of both categories are. Furthermore, attachment disorder symptoms are more common with children being exposed to abuse or neglect or being separated from prior caregivers [12, 13]. Also, children in foster care have a higher risk of showing attachment disorder symptoms [14]. This is mainly because of earlier experiences of abuse or neglect in their biological families, experiences of inadequate care in institutions, and or the separation from primary caregivers [13, 15].

### Criteria for RAD and DSED

Many issues of attachment disorders remain unclear and the diagnostic description has been criticized [14]. The criteria have been revised repeatedly [16] with major changes from DSM-IV to DSM-5 [17].

All recent DSM and ICD describe an inhibited and a disinhibited disorder. Whereas in the DSM-IV, two subtypes of attachment disorder are distinguished, in ICD-10 and DSM-5 the two patterns are two distinct disorders. The former “Reactive Attachment Disorder of Infancy and Early Childhood” now solely refers to the inhibited type of attachment disorder in the DSM-5, while the disinhibited type is reframed under the concept “Disinhibited Social Engagement Disorder”. The separation of the two disorders has been supported by several studies [5, 18].

In general, the inhibited type is characterized by withdrawal, hypervigilance and ambivalence towards the caregiver. There is neither organized attachment behavior, nor social engagement in the formation of relationships with caregivers. In contrast, children with the disinhibited type seek contact and proximity to any available person. This behavior pattern relates to indiscriminate friendliness [19].

### Assessment of attachment disorder symptoms

The validation of attachment disorders turned out to be a complex process [16] and there is no generally agreed assessment tool [12, 15, 20]. Therefore, different methods for diagnosing attachment disorders have been established in research throughout the last two decades. Regarding the diagnostic criteria and the assessment of attachment disorder, a lot of research has been done recently.

Already in 2003 O’Connor and Zeanah [21] reviewed the main approaches of assessment. Methods assessing attachment disorder symptoms include behavior observation protocols, interviews, or questionnaires. These methods have been approved in several studies since then. In a recent review Zeanah and Gleason [22] showed, that continuous as well as categorical measures are able to reliably identify RAD and DSED in samples of children at risk. The authors stated, that the constructs of RAD and DSED seems to be robust and the disorders could be diagnosed reliably using different measures within different samples.

In several studies, parent report has shown strong interrater and test–retest reliability [2, 9, 12]. Furthermore, factor analyses of the Disturbance of Attachment Interview (DAI) [23] demonstrated that the two types of disorder were distinct in a Dutch, a German as well as in a Norwegian sample of foster children [24–26]. Nevertheless, the interview addresses only one informant, thus an informant bias cannot be ruled out [27]. One diagnostic questionnaire for measuring attachment disorders has been developed by Minnis, Rabe-Hesketh and Wolkind [28]. Before that, diagnostic instruments for attachment disorders had not been very well validated, and the authors aimed to provide a method for measuring both types of attachment disorder. In a factor analysis two clusters referring to the inhibited and the disinhibited disorder could be revealed. Additionally, the questionnaire was used as a summarized measure for both disorder types [29].

Different observational settings were used to diagnose attachment disorder symptoms. Before reliable observation protocols were developed, most observations were unstructured and the reports on indiscriminate behavior in experimental settings were anecdotal [30]. Referring to several authors who used the Strange Situation Procedure (SSP) [31], a reliable laboratory measure of indiscriminate behavior was developed by Riley et al. (Rating for Infant-Stranger Engagement: RISE) [32]. The method assesses socially indiscriminate behavior during the SSP. By accounts of Lyons-Ruth et al. [30] good inter-rater reliability and a significant test–retest stability was given using the instrument, which assesses indiscriminate behavior in the same situation as attachment behavior. Another observational measure of indiscriminate behavior has been developed by Gleason et al. [9]. The Stranger at the Door (StrD) [9] assesses a child’s willingness to go off with a stranger during a standardized procedure. It was found, that socially indiscriminate behavior as measured in the StrD was associated with institutional care [33]. Furthermore, the behavior in the StrD predicted the interview-derived diagnosis in most cases [9]. To our knowledge, the procedure has not been performed by other researchers yet.

### The present study

The goal of the present study is to examine the association of four established assessment tools for attachment disorders. The sample consists of foster children who have experienced neglect and maltreatment in their biological families and were assessed at the beginning of their placement in a new long-term foster home (as reported elsewhere: [34, 35]. Four assessment approaches are implemented, evaluated and compared. Thereby, similarities of and differences between approaches should be clarified.

There is more research evidence regarding the disinhibited type of attachment disorder, respectively the DSED, and thus there exist more tools to assess this type. Just two methods used in this study are constructed to assess both the disinhibited and the inhibited symptoms, while two only refer to the disinhibited type.

Since all assessment tools are based on the definition of the attachment disorder by the DSM-IV, the results for the two disorder types should be comparable. On the other hand, the assessment tools differ a lot regarding the situations and circumstances that are assessed. Thus, the aim of this study is to explore the associations between the different measurements. Already Oliveira et al. [36] found tools for the disinhibited scale to be connected, namely the “Rating for Infant Stranger Engagement” and the “Disturbance of Attachment Interview”. Furthermore, Gleason et al. [9] demonstrated a correlation between parent report in the DAI and the Stranger at the Door procedure. Thus, it is expected that the tools for the disinhibited symptoms are linked to each other. Regarding the inhibited subtype Zeanah et al. [37] found a moderate convergence between caregiver ratings and less attachment behavior in the Strange Situation Procedure. So far only few research has been done assessing different methodological approaches and therefore it is worthwhile to examine their convergence and divergence.

## Methods

### Participants

The sample comprises 55 foster children with their current primary caregiver. The participants were recruited through German youth welfare services around Dortmund and the Ruhr valley and the Franconian cities Erlangen and Nuremberg. At the time of the examination, the children were aged between 12 and 82 months (M = 35.87; SD = 18.37) and 50.9% were female (n = 28). The children had spent 78 days on average in their present foster families. Before the current placement 87.3% (n = 48) of the infants had either lived in short-term foster families or group-home care. There had been up to 5 changes of out-of-home-placement (M = 1.25, SD = 0.93). Information about foster children’s pre-placement experiences were given by the social workers from the youth welfare department. In 83.6% of the cases (n = 46) emotional abuse had been the reason for the foster family placement. Neglect has been reported in 74.5% of the cases (n = 41). Other reasons were psychological diseases of one of the parents, physical abuse or voluntary referral of the biological parents.

In most cases (n = 48; 87.3%) the foster mother was the primary caregiver participating with the child.

### Procedure

The data presented in the current study was assessed within a longitudinal study of children in foster care at three points of measurement throughout the first year of placement (wave 1 to 3) (as reported elsewhere: [34, 35]). The current analyses include solely data of the first assessment (wave 1). At each wave, the caregiver-child dyads were observed twice within 2 weeks, once at home and once at the university. At home, foster children and their foster caregivers were observed in a semi-structured videotaped visit of 3 h. Among others, the Stranger at the Door procedure was performed and the questionnaires was given to the caregiver. During the lab visit, carried out 2 weeks after the home visit, the Strange Situation Procedure and the Disturbances of Attachment Interview took place.

### Measures

#### Rating for Infant-Stranger Engagement (RISE) [32]

The Rating of Infant Stranger Engagement is a measurement of indiscriminate attachment behaviors by evaluating attachment-related forms of engagement with the stranger by the infant during the Strange Situation Procedure. Each infant is rated on a scale from 1 to 9, evaluating the extent in which the infant accepts physical contact to the stranger and the extent of the child’s engagement with the stranger, compared to the primary caregiver. A score of 5 represents equal engagement of the child with the stranger and the caregiver, whereas scores lower than 5 indicate a preference for the caregiver and scores higher than 5 indicate non-normative forms of affective engagement with and attachment behavior towards the stranger. The rating was conducted by three trained independent raters using the original manual. The raters were blind to all other data from the study and accomplished inter-rater-coefficients of Cohen’s kappa κ = .77, κ = .80 and κ = .91.

#### Stranger at the Door (StrD) [9]

To measure indiscriminate attachment behavior a modified version of the “Stranger at the Door”-procedure as developed originally by Zeanah et al. [37] was accomplished. After prearrangements with the caregiver, both caregiver and child answered the door at the beginning of each home visit. When the caregiver opened the door, a female investigator, the child had not seen before, asked the child to follow her outside, saying “My name is […]. Would you please come along with me?” while reaching out for the child’s hand. Previously, the caregiver had been instructed not to give the child any signs. If the child left with the stranger, they walked together for about 50 m away from the house, respectively at an apartment building, they went one or two floors downstairs, and then returned. The stranger and another independent rater coded the infant’s reaction, using a specially designed observation sheet. It was coded if the child showed attachment behaviors towards the caregiver, checked back with the caregiver, clung to the caregiver, or behaved anxiously. Furthermore, it was assessed if the child followed the stranger, tried to get in contact with the stranger outside, displayed attachment behaviors during the time away, searched contact with the caregiver after return, and if the child was still in contact with the stranger after return. A sum score over four items (back checking with the caregiver, going off with the stranger, physical contact with the stranger when going outside and when returning) was conducted, giving a quantitative measure (range 0–4) of the indiscriminative social behavior. A categorical diagnosis was made when the child went off with the stranger without checking back with the caregiver. Both observers agreed in most cases (Pearson’s correlation coefficient *r *= .96, p < .01) and for the analysis a mean score of the observations was used.

#### Disturbance of Attachment Interview (DAI) [23]

The Disturbance of Attachment Interview is a semi-structured interview with the primary caregiver of the child investigating both types of attachment disorder symptoms [39]. The manual contains 12 main questions referring to the existence and extent of attachment disorder symptoms. A German translation of Smyke and Zeanah’s manual [23] was used, which had been used in a prior sample of foster children where the structure of the factors within the translation has been confirmed [25]. For each item (one to eight) it was assessed whether the described behavior is shown clearly (referred to by the score “0”), sometimes (= 1) or rarely (indicates a “2”). The items nine to twelve are scored the reverse way: not present behavior represents a 0, somewhat shown behavior a 1 and obvious existence of the described behavior is coded with a 2. Signs of emotionally withdrawn/inhibited RAD were assessed with five questions and sum scores ranging from 0 to 10, whereas signs of indiscriminately social/disinhibited DSED were measured with four items leading to a sum score ranging from 0 to 8. A high sum score thus indicates the presents of more attachment disorder symptoms. According to Zeanah et al. [27] a cut-off can be determined at three. If the sum score in one category is higher or equal to three, the score can be considered as conspicuous. Three independent native German coders coded the transcripts of the interviews. The raters were trained with a set of Portuguese cases translated into English from the lab around Isabel Soares, Portugal. The mean correlation (Pearson’s correlation coefficient) with the Portuguese ratings is *r *= .8. Further more difficult cases were discussed with the lab of Carlo Schuengel in the Netherlands and resolved by conference, similar to Zeanah et al. [38]. 30% of all interviews were coded by a second rater and inter-rater-coefficients of Cohen’s kappa κ = .76 for the inhibited scale and κ = .80 for the disinhibited scale were accomplished.

#### Reactive Attachment Disorder Questionnaire [28]

The questionnaire for reactive attachment disorders contains 17 items referring to symptoms of attachment disorders of both types, coded on a scale from 0 to 3. Both attachment disorder types are not referred to separately and overall mean scores from 0 to 51 can be achieved, with high scores indicating more symptoms of attachment disorders. Minnis et al. [28] could show a satisfying internal consistency and test–retest reliability. As the German version of the questionnaire does not provide a valid factor structure or a cut-off, the sum score is used with high scores indicating more symptoms of RAD.

## Results

### Descriptive results

Pearson’s correlation coefficients were conducted to examine the interrelation between the age of the child as well as the length of placement and attachment disorder symptoms. We found no significant correlation between the duration of placement and the disinhibited attachment disorder symptoms in the DAI (*r *= − .11, ns.), the RISE (*r *= − .02, ns.), the SatD (*r *= − .23, ns) or the sum score in the RAD Questionnaire (*r *= .28, p < .05). A correlation between the time in the foster family and the inhibited scale of the DAI (*r *= .28, p < .05) could be found. Regarding the age of the foster children, small correlations to both scales of the DAI (*r *= .27 and *r *= .30, p < .05), the RISE (*r *= .34, p < .05) and the RAD Questionnaire (*r *=.28, p < .05) are found. There was no significant correlation between the children’s age and the SatD score (*r *= .08, ns.).

The descriptive values of all measures are shown in Table 1.

**Table 1:** Descriptive values of all measures

	RISE	StrD	DAI disinhibited	DAI inhibited	RAD Questionnaire
N	55	55	55	55	55
M (SD)	3.99 (1.20)	1.30 (1.23)	1.90 (1.77)	.83 (.96)	18.14 (5.03)
Diagnosis frequency	3	23	17	3	–

#### Rating for Infant-Stranger Engagement

In total, 11.3% of the infants (n = 6) were seeking some kind of physical contact with the stranger. Referring to the cut-off score set by Riley et al. [32] 94.5% of the children (n = 52) showed more or equal engagement with the mother compared to the stranger, while 5.5% (n = 3) reached a score higher than five, which indicates non-normative attachment behavior towards the stranger.

#### Stranger at the Door

The procedure was used with all participants. Overall 58.2% of the infants (n = 32) agreed to go off with the stranger while the remaining children (n = 23) negated the invitation. Following the request 30.9% of the children (n = 17) contacted the caregiver whether verbally or through eye contact. The children who went off with the stranger showed no back-checking behavior in most of the cases (71.9%; n = 23). The mean of the conducted sum score was 1.30 (SD = 1.23) with a range from 0 to 3.

#### Disturbance of Attachment Interview

The sample showed elevated scores on both scales assessed by the DAI. Referring to the cut-off score, 5.5% (n = 3) of the sample showed an impairment in the inhibited category of attachment disorders. For the disinhibited scale 17 cases (30.9%) scored above 3.

#### Reactive Attachment Disorder Questionnaire

The following analysis refer to the total sum score as a representation of overall attachment disorder related behavior, in accordance to Minnis et al. [27]. The mean of the sum score was 18.14 (SD = 5.03), with a range from 6 to 32.

### Examination of correlations between the approaches

Pearson’s correlation coefficients were conducted for all measures. The inter correlations are displayed in Table 2. It shows that the three measures that assessed disinhibited attachment disorder symptoms correlated significantly. The RISE score, the StrD sum score and the disinhibited scale of the DAI significantly correlated with each other.

**Table 2:** Pearson’s correlation coefficients over all measures

	StrD	DAI disinhibited	DAI inhibited	RAD Questionnaire
RISE	.40**	.41**	.19	.10
StrD		.42**	.06	.04
DAI disinhibited			.28^+^	− .02
DAI inhibited				.40*

^+^ p < .05, * p < .005, ** p < .001

Furthermore, there was a significant correlation between the RAD Questionnaire score and the inhibited scale of the DAI.

The association of all disinhibited measures that can be used for a categorical diagnosis was examined. All diagnosis frequencies are displayed in Table 3. Regarding the RISE, the small number of children that were diagnosed categorically is noticeable. Of the children who were diagnosed as disinhibited in the DAI, 70.6% went off with the stranger at the StrD. Conducting the Chi square test, the results of the disinhibited DAI scale were associated with the behavior in the Stranger at the Door procedure, χ^2^(1, N = 55) = 8.37, p < .05. 71.1% of the children that had no disinhibited diagnosis in the DAI did not leave with the stranger at the StrD and 94.7% of those children did not hit the cut-off score in the RISE. 57.7% of the children that did not hit the cut-off score in the RISE, did not leave with the stranger in the StrD.

**Table 3:** Frequencies of overlapping diagnoses using the categorical measures and percentage values row-wise

	Going off without checking back		RISE above cut-off	
	Yes (23)	No (32)	Yes (3)	No (52)
DAI disinhibited disorder				
Yes (17)	12 (70.6%)	5 (29.4%)	1 (5.9%)	16 (94.1%)
No (38)	11 (28.9%)	27 (71.1%)	2 (5.3%)	36 (94.7%)
RISE above cut-off				
Yes (3)	1 (33.3%)	2 (66.7%		
No (52)	22 (42.3%)	30 (57.7%)		

## Discussion

In the present study four approaches for determining symptoms of attachment disorders have been accomplished in a sample of foster children. All approaches aimed at assessing attachment disorder symptoms. Still the amount of conspicuousness is not similar within the different measurements.

Interestingly, only the inhibited symptoms as measured with the DAI showed an interrelation with the time the child had already spend in the foster family before the first assessment. Since the inhibited symptoms have an internalizing character, they might be mistaken for shyness at first. Maybe it takes more time for caregivers to identify inhibited attachment disorder behavior as such, with more reported symptoms after more time spend in foster placement. On the other hand, the children’s age slightly interrelated with all measures except with the Stranger at the Door procedure. Due to the fact, that a higher age at placement represents more time in potentially pathologic care of the prior family or multiple placements before the current foster care, more symptomatic behaviors are non-surprising. Consistently, Smyke et al. [40] demonstrated that early placement in foster care was associated with fewer disinhibited attachment disorder symptoms, compared to children who remained institutionalized longer.

### Comparison with other samples

The Rating for Infant Stranger Engagement (RISE) shows the current sample to be less conspicuous than the high-risk sample of maltreated children examined by Lyons-Ruth et al. [30]. Also, regarding the cut-off only a minority of the children were categorized. The findings for the Stranger at the Door are remarkable: more than half of the children agreed to go off with the stranger in the procedure. The total amount of children who left with the stranger in our procedure is thus much higher than found for example by Gleason et al. [9]. In most cases the children went off without checking back with their caregivers. An explanation might be that the stranger in our study appeared to be very friendly and in particular motivated the child to go off with her. The sum scores for the Disturbances of Attachment Interview in the inhibited and the disinhibited category are below those found by Zeanah et al. [38] for institutionalized but are higher than those for never institutionalized children. The mean score for the disinhibited type is best comparable to Gleason et al. [9]. The total number of children showing symptoms of RAD is twice as high as found by Oosterman and Schuengel [41]. There was a high variance within the sum scores of this sample.

Overall the descriptive results assort the state of research. The recent sample has not experienced the same amount of deprivation children in Romanian institutions have been exposed to [3, 4]. Thus, the recent sample is less affected by RAD symptoms.

### Examination of the assessments

The results show a strong correlation between the continuous measurements for disinhibited attachment disorder symptoms. The RISE procedure aims at assessing the core characteristics of the disinhibited attachment disorder: contact seeking to any available caregiver and indiscriminate behavior. These behavior patterns are similar to what the “Stranger at the Door” procedure focuses on. Both approaches are based on a behavioral observation. The connection between these two measurements is thus according to expectations. Consistent to the findings from Oliveira et al. [36] the disinhibited attachment disorder symptoms measured by the RISE are correlated with the disinhibited scale of the DAI. Among others, the DAI scale for the disinhibited disorder already includes the question whether the child would be willing to go off with a stranger. The link between the disinhibited scale in the DAI and going off in the StrD is thus according to expectations and consistent with Gleason et al. [9]. In summary, the findings show reasonable links between the three continuous measures assessing the disinhibited type of attachment disorders (RISE, StrD and DAI).

Regarding the categorical approach of these measures it is noticeable, that very few children are categorized with the RISE cut-off score and a lot of children went off with the stranger. Regarding to the sensitivity and specificity of the measures it is noticeable, that more than two-thirds of the children showing signs of disinhibition in the DAI were also willing to go off with the stranger in the StrD, whereas two-thirds that showed no disinhibited signs in the DAI did not leave with the stranger. This association was proved to be significant. Gleason et al. [9] even found concordance of the two measures in about 85% of the cases. Children that did not hit the RISE cut-off, were in more than two-thirds also not diagnosed in the DAI and more than half of the children did not leave with the stranger in the StrD. The association of the categorical measures of DSED is less obvious and leaves more variance. The measures differ regarding the sensitivity and specificity. Whereas the RISE seems to underestimate the indiscriminate behavior the StrD overestimated the disinhibited attachment disorder in this sample.

The two methods including inhibited symptoms are only moderately connected, too (DAI and RAD Questionnaire) even though both measurements are based on the same source of information (primary caregiver). A reason might be the internalizing character of the inhibited patterns, which makes the observation more complicated. Furthermore, the questionnaire did not assess inhibited symptoms separately.

In accord with Minnis et al. [28] the version of the RAD Questionnaire failed to represent the two different scales of attachment disorder (inhibited and disinhibited) in the present data. Since the two types are separated categories in the DSM-5, a summarized sum score seems to be no longer useful for the diagnosis.

### Strengths, limitations and further directions

One limitation of the present study might be the small sample size. The participation was also voluntary, thus the results may not be generalized. The results still seem to be useful as this population is not so easy to access and any kind of standardized information helpful.

In the sample the StrD overestimates the presence of disinhibited behavior. As discussed, a proper explanation might lie within the provided setting presenting a very friendly female stranger. The conducted sum score seems to be more sensitive in this sample, because it assesses more selective attachment behaviors than solely going off with the stranger or not.

The RISE compares behavior towards strangers to behavior towards the primary caregiver. A child’s general tendency to seek or to avoid contact is thus considered. A point of criticism is due to the fact that the Strange Situation Procedure is used for assessment of attachment disorder behavior, because the SSP is based on the assumption that there is an attachment relationship between the child and the caregiver which is meant to be assessed [21]. Besides, the procedure is relatively short and there is only one observed situation that leads to a diagnosis. On the other hand, the standardized setting including a stranger and the separation within the situation provide interesting opportunities for the examination of the child’s interactive behaviors. Furthermore, some of the children that participated were relatively old. The small interrelation found between age of the child and seeking contact to the stranger in the RISE might be seen as a result of more approaching behavior of older children. Adequate behavior towards a stranger change with age, and thus the RISE might not be sensitive to the attachment disorder behaviors of older children.

Minnis et al. [28] developed the RAD Questionnaire for a sample of foster children aged between 5 and 16. Most of the children in this sample are younger than 5 and thus it is questionable, if the questionnaire can be used within this sample. Closer examination of the items shows, that most assessed behaviors are not necessarily related to age and can be interpreted related to age, such as “tends to be afraid of new things or situations”, “is too friendly with strangers”, “is demanding or attention seeking” or “when you have been parted for a short time, he/she is happy to see you”. Other items are less suitable for toddlers. For example, “very clingy/wants to be with you all the time” describes a lot of toddlers and is not necessarily a symptomatic behavior. Furthermore, items like “has few friends” and “often starts conversations” are difficult to assess in children younger than 5. Overall, the age of the sample might have negatively influenced the results and there remain questions regarding the interpretation of the RAD Questionnaire. Most research focuses on the disinhibited type, which is much more obvious because the related behavior patterns are externalizing. The inhibited patterns are internalizing and less obvious. This might be a reason for the lack of diagnostic tools regarding the inhibited type. At the state of the research, parent report (as in the DAI) seems to be the most trustful assessment method. Although the DAI suffers from a potential rater bias, the interview contains questions with regard to different situations and gives the possibility to describe a certain behavior according circumstances.

## Conclusions

Overall, attachment disorders could be diagnosed with several tools within this sample and according to other authors [9, 12]. Since each assessment contains a special point of view on the syndrome the results must be interpreted in consideration of the special focus of the used method. Zeanah et al. [5] stated several recommendations for clinicians diagnosing attachment disorders. It is suggested to include current behavior patterns as well as the information regarding the history of attachment behavior. Furthermore, an observational paradigm and comprehensive psychiatric assessment is recommended. Therefore, for a valid diagnosis, a multi-methodical approach is recommended.

Already the fact that the attachment disorders were defined differently in DSM-IV and ICD-10 [42] conveyed that the classification lacked further investigation [42, 43]. With the review of the category for DSM-5 [17] an important step in the direction of coherent understanding of the distortions is made. For the first time the inhibited and the disinhibited category are separated according to both DSM and ICD. As a result, the categories may no longer be considered as two types of the same distortion. Thus, in the diagnosis it is important to assess the two disorders separate.

## Abbreviations

RAD: reactive attachment disorder
DSED: disinhibited social engagement disorder
DAI: Disturbance of Attachment Interview
SSP: Strange Situation Procedure
RISE: Rating for Infant Stranger Engagement
StrD: Stranger at the Door
